# Epigenetic Modulation of Gremlin-1/NOTCH Pathway in Experimental Crescentic Immune-Mediated Glomerulonephritis

**DOI:** 10.3390/ph15020121

**Published:** 2022-01-20

**Authors:** Lucia Tejedor-Santamaria, Jose Luis Morgado-Pascual, Laura Marquez-Exposito, Beatriz Suarez-Alvarez, Raul R. Rodrigues-Diez, Antonio Tejera-Muñoz, Vanessa Marchant, Sergio Mezzano, Carlos Lopez-Larrea, Anna Sola, Gema Maria Fernandez-Juarez, Alberto Ortiz, Sandra Rayego-Mateos, Marta Ruiz-Ortega

**Affiliations:** 1 Cellular Biology in Renal Diseases Laboratory, IIS-Fundación Jiménez Díaz, Universidad Autónoma Madrid, 28040 Madrid, Spain; lucia.tejedor@quironsalud.es (L.T.-S.); jmorgado@uco.es (J.L.M.-P.); laura.marqueze@quironsalud.es (L.M.-E.); antoniotemu@gmail.com (A.T.-M.); vmarchant.hernandez@gmail.com (V.M.); srayego@fjd.es (S.R.-M.); 2 REDINREN Instituto de Salud Carlos III, 28029 Madrid, Spain; bea230@hotmail.com (B.S.-A.); RRodriguez@fjd.es (R.R.R.-D.); inmuno@hca.es (C.L.-L.); asola@idibell.cat (A.S.); gema.fernandezjuarez@salud.madrid.org (G.M.F.-J.); aortiz@fjd.es (A.O.); 3 Department of Immunology, Hospital Universitario Central de Asturias, 33011 Oviedo, Spain; 4 School of Medicine, Universidad Austral de Chile, Valdivia 5090000, Chile; mezzano.sergioa@gmail.com; 5 Department of Experimental Nephrology, Institut d’Investigació Biomèdica de Bellvitge (IDIBELL), 08908 Barcelona, Spain; 6 Unidad de Nefrología, Hospital Universitario Fundación Alcorcón, 28922 Madrid, Spain; 7 Division of Nephrology and Hypertension, IIS-Fundación Jiménez Díaz, Universidad Autónoma Madrid, 28040 Madrid, Spain

**Keywords:** crescentic glomerulonephritis, chronic kidney disease, bromodomain, BET proteins, Gremlin, NOTCH

## Abstract

Crescentic glomerulonephritis is a devastating autoimmune disease that without early and properly treatment may rapidly progress to end-stage renal disease and death. Current immunosuppressive treatment provides limited efficacy and an important burden of adverse events. Epigenetic drugs are a source of novel therapeutic tools. Among them, bromodomain and extraterminal domain (BET) inhibitors (iBETs) block the interaction between bromodomains and acetylated proteins, including histones and transcription factors. iBETs have demonstrated protective effects on malignancy, inflammatory disorders and experimental kidney disease. Recently, Gremlin-1 was proposed as a urinary biomarker of disease progression in human anti-neutrophil cytoplasmic antibody (ANCA)-associated crescentic glomerulonephritis. We have now evaluated whether iBETs could regulate Gremlin-1 in experimental anti-glomerular basement membrane nephritis induced by nephrotoxic serum (NTS) in mice, a model resembling human crescentic glomerulonephritis. In NTS-injected mice, the iBET JQ1 inhibited renal Gremlin-1 overexpression and diminished glomerular damage, restoring podocyte numbers. Chromatin immunoprecipitation assay demonstrated BRD4 enrichment of the *Grem-1* gene promoter in injured kidneys, consistent with Gremlin-1 epigenetic regulation. Moreover, JQ1 blocked BRD4 binding and inhibited *Grem-1* gene transcription. The beneficial effect of iBETs was also mediated by modulation of NOTCH pathway. JQ1 inhibited the gene expression of the NOTCH effectors *Hes-1* and *Hey-1* in NTS-injured kidneys. Our results further support the role for epigenetic drugs, such as iBETs, in the treatment of rapidly progressive crescentic glomerulonephritis.

## 1. Introduction

Chronic kidney disease (CKD) is defined as abnormalities in kidney structure or function, present for >3 months and with implications for health [1]. CKD is projected to become the fifth global cause of death by 2040 [2], revealing the importance of research in this area [3]. Glomerular kidney disease cause 18% of CKD cases requiring kidney replacement therapy, illustrating the failure of current therapeutic approaches to prevent CKD progression to end-stage renal disease (ESRD) [4]. Rapidly progressive glomerulonephritis (RPGN) is one of the most aggressive forms of CKD, characterized by the proliferation of intrinsic glomerular cells in the Bowman’s space, leading to the formation of crescents that evolve to fibrotic structures and loss of functioning glomeruli [5,6]. Thus, the underlying structural kidney damage for the clinical presentation, termed RPGN, is crescentic glomerulonephritis (cGN). Most GNs are immune-mediated, related to the presence of anti-neutrophil cytoplasmic antibodies (ANCA), anti-glomerular basement membrane (anti-GBM) antibodies or local immune-complex deposition. Most patients are treated with immunosuppressive therapy, but the residual risk of ESRD remains high despite treatment [2].

Epigenetic mechanisms, especially DNA methylation and post-translational histone modifications, are dynamic processes that differentially regulate gene expression in normal and diseased states [7]. Novel approaches targeting protein–protein interactions of epigenetic ‘readers’ have emerged as a novel source of therapeutic drugs [8]. The protein members of the bromodomain (BRD) and extraterminal (BET) family (including BRD2, BRD3, BRD4 and BRDT) recognize acetylated lysine residues on core histones and regulate gene transcription through the recruitment of coactivator proteins involved in transcriptional initiation and elongation, acting as epigenetic ‘readers’ [9]. BET proteins can also bind to acetylated lysine residues on transcription factors, therefore regulating gene transcription [10]. Selective inhibitors of BET proteins (iBETs), such as the small molecule JQ-1, displace bromodomain binding to acetyl-lysine residues on histones and non-histones proteins [11,12]. Different iBETs have protected mice from malignancy and experimental inflammation, including from kidney diseases [13].

ANCA-associated vasculitis is the major cause of RPGN [5,14,15]. We have recently described that Gremlin-1 is a potential urinary biomarker of human ANCA-associated cGN [16]. High urinary levels of Gremlin-1 were associated with a more severe disease activity as represented by the number of glomerular crescents, tubulointerstitial fibrosis and interstitial inflammation. Gremlin-1 belongs to the cysteine knot superfamily of proteins, and it has been proposed as a biomarker and a therapeutic target in experimental renal diseases [17,18,19]. Preclinical studies have demonstrated that Gremlin-1 activated several downstream pathways, including nuclear factor-κB (NF-κB) and NOTCH, linked to inflammation in the kidney [20,21,22,23]. Many studies have described that the NOTCH signaling pathway is activated in human CKD, including glomerular diseases such as diabetic kidney disease (DKD), IgA nephropathy and focal segmental glomerulosclerosis (FSGS) [24,25,26,27,28]. The activation of NOTCH is mediated by successive proteolytic cleavages of the NOTCH receptor which release its intracellular domain (NICD) from the cellular membrane. Then, NICD is translocated into the nucleus, where it forms a nuclear complex with the transcriptional activator RBP-Jκ (Recombination Signal-binding Protein 1 for J-kappa) and then activates the transcription of downstream NOTCH target genes, including the effectors of this pathway Hes (hairy and enhancer of split) and Hey (Hes-related proteins) [28,29]. In vivo, the administration of recombinant Gremlin-1 into murine kidneys increased NICD nuclear levels and upregulated *Hes-1* gene expression. Moreover, NOTCH inhibition blocks several deleterious actions of Gremlin-1 in the kidney in vivo and in vitro [20,22,23], illustrating the complex relation between Gremlin-1 and the NOTCH pathway.

The model of anti-glomerular basement membrane (GBM) induced by nephrotoxic serum (NTS) administration in mice is an experimental model commonly used to study human cGN [30,31]. We have recently observed that in NTS-injected mice, treatment with JQ1 restored the changes in renal function, ameliorated glomerular lesions and diminished renal inflammatory cell infiltration by mechanisms that involved the direct inhibition of several proinflammatory genes, as well as the blockade of the NF-κB pathway’s activation and, therefore, the subsequent transcription inhibition of related-genes [32].

The aim of this paper was to evaluate whether iBETs could regulate Gremlin-1 in NTS mice, trying to define the final targets of bromodomain/acetyl-lysine binding, subsequent molecular interactions and gene transcription regulation, with special attention to the NOTCH pathway. This study could help to develop better therapeutic tools for the treatment of cGN.

## 2. Results

### 2.1. BET Inhibition Ameliorates Renal Damage in Experimental Nephrotoxic Nephritis

**Figure 1:**
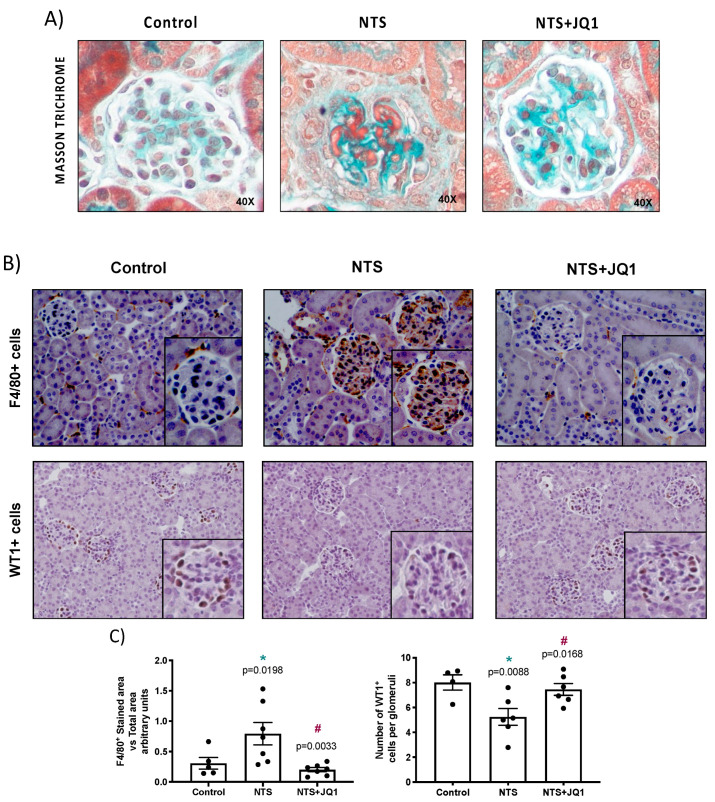
JQ1 diminishes renal damage in nephrotoxic nephritis. Glomerulonephritis was induced in C57Bl/6 mice by the administration of NTS, and mice were studied 10 days later. Mice were treated daily with JQ1 (100 mg/kg body weight per day) or vehicle, starting before the first NTS-injection. In paraffin-embedded kidney sections, renal morphology was evaluated by (**A**) Masson trichrome staining and (**B**) immunohistochemistry with specific antibodies. JQ1 ameliorated glomerular damage (**A**), diminished glomerular monocyte inflammatory cell infiltration (F4/80^+^ monocytes/macrophages/dendritic cells) and restored healthy podocyte number (WT1^+^ cells) (**B**) to values similar to control untreated mice. (**C**) Quantification of immunohistochemistry staining (F4/80+ and glomerular WT1^+^ cells). Data are expressed as the mean ± SEM of 5–7 animals per group. * *p* < 0.05 vs. control; ^#^ *p* < 0.05 vs. NTS-injected mice. Panel (**B**) shows a representative animal from each group (200× magnification), including a detail of glomeruli.

The model of NTS administration in mice is commonly used to study mechanisms of human cGN. This model is characterized by the proliferation of intrinsic glomerular cells in the Bowman’s space, mesangial fibrosis and podocyte damage, resulting in the rapid loss of renal function, resembling the human disease [30]. We previously described that JQ1 ameliorates impaired renal function and glomerular lesions in this model [32] (Figure 1A), mainly inhibiting extracapillary proliferation and fibrinoid necrosis in glomerular cells, as previously described [32], although crescent formation was not found at this earlier time point.

Importantly, NTS-injured kidneys also presented macrophage infiltration that was significantly diminished by JQ1 treatment (Figure 1B). To further evaluate glomerular damage, healthy podocytes that express Wilms’ tumor protein 1 (WT-1) were evaluated. NTS administration in mice resulted in a dramatic loss of glomerular WT1^+^ cells, which were restored by JQ1 (Figure 1B).

### 2.2. Gremlin-1 Is Overexpressed in Experimental Nephrotoxic Nephritis in Mice: Effect of BET Inhibition

**Figure 2:**
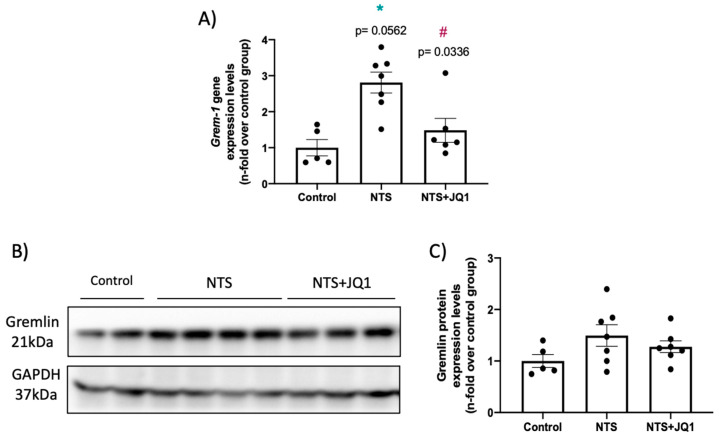
JQ1 diminishes renal Gremlin-1 overexpression at the gene and protein levels in nephrotoxic nephritis. NTS mice were treated daily with JQ1 (100 mg/kg body weight per day) or vehicle. (**A**) RNA was isolated from frozen whole kidney samples, and *Grem-1* gene expression levels were evaluated by real-time qPCR. (**B**) Protein levels of Gremlin-1 were quantified by Western blot in total kidney extracts. Figure B show a representative blot of 2 to 4 mice per group. GAPDH was used as loading control. (**C**) Quantification of Gremlin protein levels. Data are expressed as the mean ± SEM of 5–7 animals per group. * *p* < 0.05 vs. control; ^#^ *p* < 0.05 vs. NTS-injected mice.

Kidney injury in mice with NTS nephritis is characterized by the upregulation of Gremlin-1 at the gene and protein levels compared to healthy control mice (Figure 2A–C). The treatment with JQ1 significantly diminished *Grem-1* mRNA levels (Figure 2).

### 2.3. Gremlin-1 Is One of the Specific Targets of BET Inhibition

**Figure 3:**
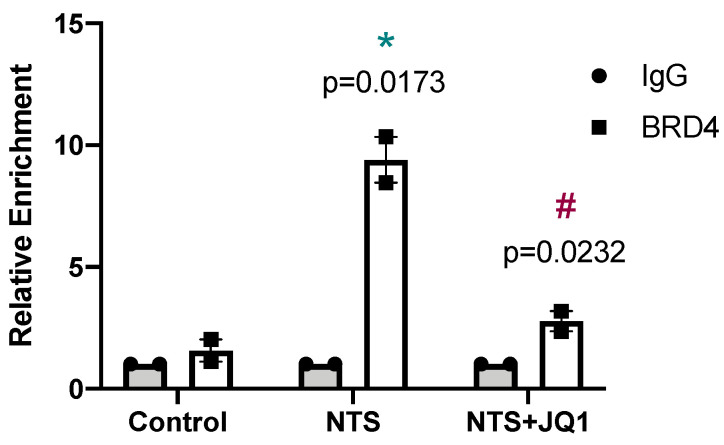
*Grem-1* is a direct target of iBETs. ChIP assays were performed in renal samples using an antibody specific for BRD4 or normal rabbit IgG, the latter being a negative control. Enrichment of BRD4-binding regions in the promoter of mouse *Grem-1* was quantified by qPCR using specific primers. In each group, samples were pulled (4–7 mice per group). Data are from two independent experiments and each qPCR was run in triplicate. Results are expressed as the *n*-fold enrichment of anti-BRD4 antibody relative to the negative control antibody (considered to be 1). * *p* < 0.05 vs. control; ^#^ *p* < 0.05 vs. NTS-injected mice.

Chromatin immunoprecipitation (ChIP) assays demonstrated that BRD4 binding to the promoter region of *Grem-1* was increased in NTS-injured kidneys compared to the controls. However, BRD4 enrichment was nearly abolished in JQ1-treated mice (Figure 3). Moreover, JQ1 inhibited the recruitment and binding of BRD4 to this promoter region and therefore reduced the transcription of *Grem-1*. These results demonstrate that BRD4 directly binds to the regulatory region of *Grem-1* promoting its gene expression and clearly shows that *Grem-1* is a direct target of JQ1.

### 2.4. JQ1 Inhibits the NOTCH Pathway in Experimental Nephrotoxic Nephritis

**Figure 4:**
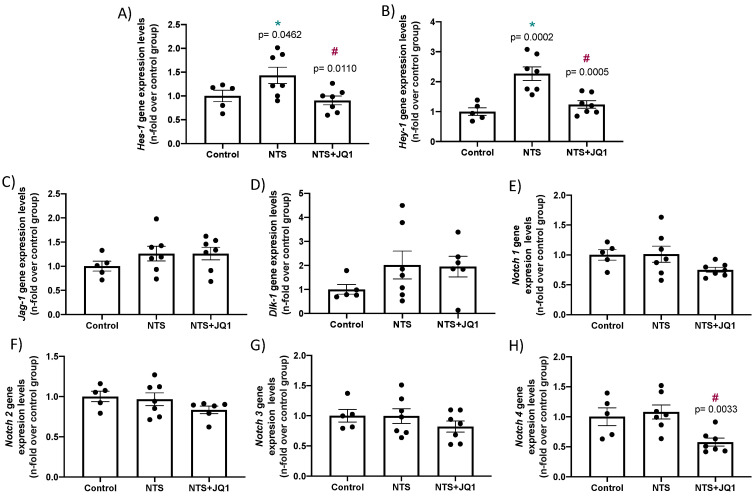
JQ1 inhibited NOTCH signaling pathway activation in experimental nephrotoxic nephritis. NTS mice were treated daily with JQ1 (100 mg/kg body weight per day) or vehicle. (**A**) RNA was isolated from frozen samples of whole kidney, and *Hes-1* (**A**) *Hey-1*, (**B**) *Jagged-1* (**C**), *Dlk-1* (**D**), *Notch 1* (**E**), *Notch 2* (**F**), *Notch 3* (**G**) and *Notch 4* (**H**) gene expression levels were evaluated by real-time qPCR. Data are expressed as the mean ± SEM of 5–7 animals per group. * *p* < 0.05 vs. control; ^#^ *p* < 0.05 vs. NTS-injected mice.

NOTCH-1 signaling pathway activation is associated to changes in the receptors, ligands and final effector expression levels of several NOTCH components in preclinical studies and in human samples [24,25,26,27,28]. Real-time PCR showed the upregulation of the downstream NOTCH-target genes *Hes-1* and *Hey-1* in injured kidneys of NTS mice compared to healthy controls (Figure 4A,B). In contrast, there were no differences in the expression of the NOTCH-1 canonical ligand and activator, *Jagged-1* involved in experimental kidney fibrosis [33,34], and in the mRNA levels of the noncanonical ligand *Dlk-1*, an endogenous inhibitor re-expressed in kidney diseases [35] (Figure 4C-D). There were also no differences in the expression levels of NOTCH receptors *Notch-1*, *Notch-2*, *Notch-3* and *Notch-4* between the NTS-injured and control kidneys (Figure 4E-H). Importantly, in NTS mice, treatment with JQ1 inhibited the gene overexpression of the NOTCH effectors *Hes-1* and *Hey-1* (Figure 4A,B).

### 2.5. Differential Gene Expression in Experimental Nephrotoxic Nephritis: Impact of BET Inhibition

**Figure 5:**
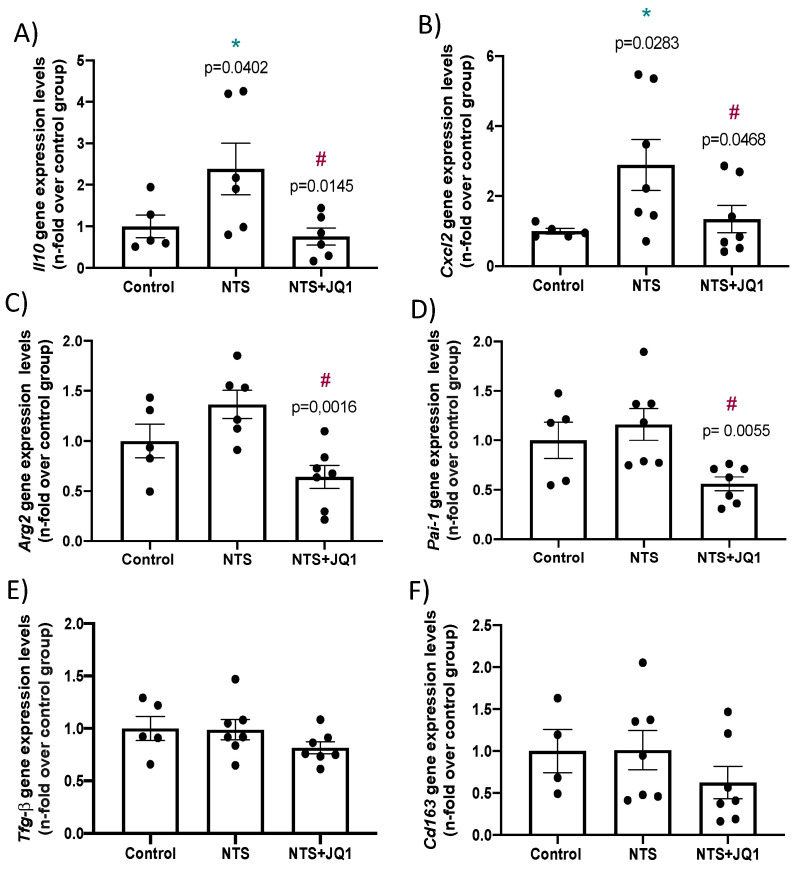
Effect of BET inhibition on gene expression in nephrotoxic nephritis. RNA was isolated from frozen samples of whole kidney, *IL10* (**A**) *Cxcl2*, (**B**) *Cd163* (**C**), *Arg2* (**D**), *Tgf-β* (**E**) and *Pai1* (**F**), and gene expression levels were evaluated by real-time qPCR. Data are expressed as the mean ± SEM of 5–7 animals per group. * *p* < 0.05 vs. control; ^#^ *p* < 0.05 vs. NTS-injected mice.

In a previous study, we demonstrated that JQ1 inhibited the renal expression of the proinflammatory factors *Ccl-2*, *Ccl-5* and *Il-6* in NTS-mice by the inhibition of BRD4 binding to their promoter regions [32]. To extend these findings, we have now evaluated whether BET inhibition can modulate other proinflammatory and profibrotic factors in NTS mice. The gene expression of *Il10* and *Cxcl2* were markedly upregulated in injured kidneys and significantly diminished by JQ1 treatment (Figure 5A,B). The M1 macrophage marker, *Arg2*, and the profibrotic factor *Pai-1* gene expression levels were significantly inhibited by JQ1, whereas they were only slightly increased in injured kidneys (Figure 5C,D). In contrast, the profibrotic factor *Tgf-β1* and the M2 macrophage marker *Cd163* were not changed in any of the groups studied (Figure 5E,F).

### 2.6. BET Inhibition Diminished the Renal Expression of the Chemokine Ccl-8 in Experimental Anti-glomerular Basement Membrane Nephritis

**Figure 6:**
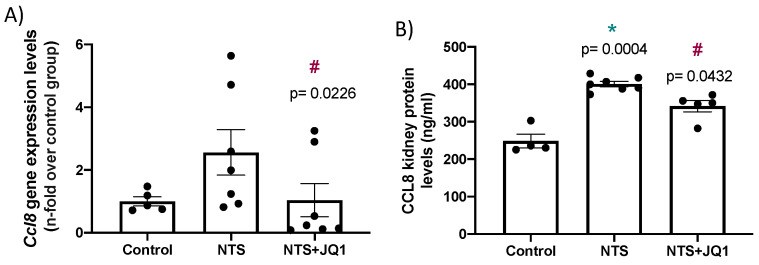
JQ1 diminishes kidney CCL8 levels in the nephrotoxic nephritis. NTS mice were treated daily with JQ1 (100 mg/kg body weight per day) or vehicle. RNA and proteins were isolated from frozen samples of whole kidney. (**A**) *Ccl8* gene expression levels were evaluated by real-time qPCR, and (**B**) CCL8 protein levels were determined by ELISA. Data are expressed as the mean±SEM of 5–7 animals per group. * *p* < 0.05 vs. control; ^#^ *p* < 0.05 vs. NTS.

The chemokine (C-C motif) ligand 18 (CCL18) has been recently proposed as a potential biomarker of human cGN [36]; therefore, we have evaluated whether JQ1 could also modulate the renal expression of *Ccl8*, the murine functional human CCL18 homologue [37,38], in the NTS model. Renal *Ccl8* gene expression was upregulated in NTS mice. Importantly, *Ccl8* mRNA levels were significantly diminished by JQ1 treatment (Figure 6A). Accordingly, CCL8 protein levels, assayed by ELISA, were significantly elevated in the kidneys of NTS mice and significantly inhibited by JQ1 (Figure 6B).

### 2.7. BET Inhibition Downregulates Gremlin-1 Expression in Other Models of Renal Fibrosis

**Figure 7:**
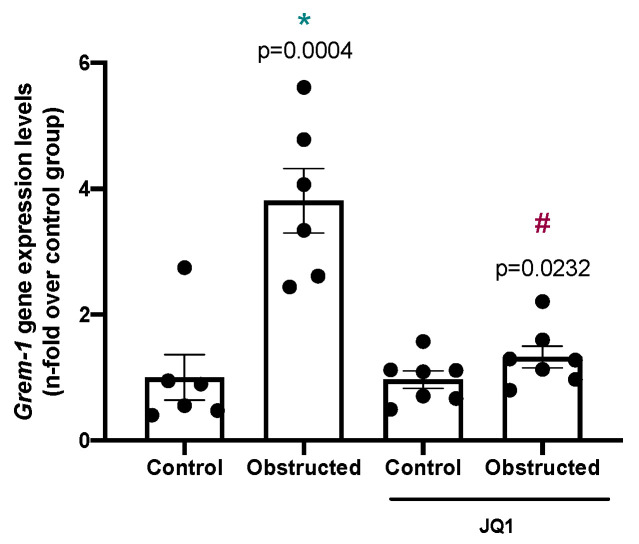
JQ1 diminishes Gremlin-1 in an experimental model of progressive tubulointerstitial fibrosis. Unilateral ureteral obstruction (UUO) was performed in C57Bl/6 mice as described in Methods, and kidneys were studied after 5 days. Some mice were treated with JQ1 (100 mg/kg body weight per day, i.p. starting 24 h before UUO). RNA was obtained from total renal extracts and *Grem-1* mRNA levels were determined by real-time qPCR. Data are expressed as the mean ± SEM of 5–7 animals per group. * *p* < 0.05 vs. control; ^#^ *p* < 0.05 vs. UUO.

In human cGN, Gremlin-1 overexpression has been described in glomerular and tubulointerstitial areas [39]. We have further evaluated whether BET inhibition can modulate Gremlin-1 in other experimental models of progressive kidney disease. To this aim, we studied the model of unilateral ureteral obstruction (UUO). This model is characterized by tubulo-interstitial fibrosis, observed as early as 5 days following renal injury, as well as Gremlin-1 overexpression in the obstructed kidneys [22,35]. We have previously described that JQ1 ameliorates renal inflammation in this model [32]. Treatment with JQ1 significantly diminished *Grem-1* gene overexpression in obstructed kidneys compared to untreated, obstructed ones (Figure 7).

## 3. Discussion

The main finding of this paper is the description of Gremlin-1 as a specific target of BET inhibition in the experimental NTS model, a mice model that resembles human progressive cGN. We have recently described that Gremlin-1 is a potential urinary biomarker of human ANCA-positive GN [16]. The current therapy for cGN is limited to harsh immunosuppression, and the majority of patients still progress towards ESRD and death, remarking an unmet medical need for novel therapeutic approaches. Our data support future research to evaluate whether iBETs could be a therapeutic option for cGN.

Selective iBETs, such as JQ1, block the interaction between BET proteins and acetylated proteins, including histones and transcription factors [12]. Early studies using iBETs have demonstrated protective effects in malignancy, dependent on the inhibition of proto-oncogene transcription and the modulation of cell proliferation [40,41]. More recently, iBETs have shown beneficial responses in a wide range of inflammatory diseases [11], mainly through the modulation of the NF-κB pathway and blocking downstream proinflammatory-related gene expression [12], as well as the inhibition of the Th17/IL17 immune response [42,43], as we have previously described in different models of experimental kidney diseases, including the NTS nephritis model [32]. Now, we have found that JQ1 inhibited the Gremlin-1/NOTCH signaling pathway, thus identifying novel mechanisms involved in the beneficial effects of iBETs in experimental kidney diseases. Moreover, chromatin immunoprecipitation assays demonstrated that JQ1 modulates BRD4 binding to the promoter region of the *Grem-1* gene, thus reducing *Grem-1* transcription, showing that this gene is a specific target of BET inhibition and clearly demonstrating the epigenetic regulation of Gremlin-1 during experimental NTS.

Gremlin-1 has been proposed as a mediator and potential therapeutic target in CKD [16,17,18,19]. The de novo *GREM1* expression has been described in several human kidney diseases, including RPGN, DKD and transplant rejection [39,44,45]. Experimental gain- and loss-of-function studies targeting *Grem-1* in renal cells as well as in murine models of kidney disease have demonstrated the key role of Gremlin-1 in the regulation of inflammation and fibrosis-related processes [22,46,47,48,49,50,51,52,53,54,55,56]. Moreover, the administration of recombinant Gremlin-1 to mice caused an early kidney inflammatory response [20]. In addition, RNA sequencing analysis of Gremlin-effects in the kidney revealed changes in biomarkers of kidney damage and wound healing pathways [57]. In human ANCA-associated cGN, Gremlin-1 is overexpressed in glomerular crescents, tubular cells and infiltrating interstitial cells [39], and it has been proposed as a potential urinary biomarker [16]. CCL18 is another potential biomarker of disease activity in ANCA-associated cGN [36]. This chemokine is one of the most highly expressed in human chronic inflammatory diseases, including allergies, fibrotic disorders and certain cancers [58], and it is highly secreted by M2 macrophages [59]. The functional analogue of human CCL18 is the mouse *Ccl8*, and they share CCR8 as a functional receptor [37,38]. Interestingly, CD163+/CCL18 expressing macrophages colocalized with Gremlin-1 protein expression in ANCA-associated cGN patients [16]. CD163 is another marker of M2-macrophages [37,60] and soluble urinary CD163 can be a potential biomarker of macrophage activation in different diseases, including cGN [37]. In ANCA-associated cGN, urinary CD11b+ and CD163+ correlated with leukocyte recruitment in the kidney [61], and urinary CD163 was a biomarker of active renal vasculitis and relapse [62]. We now show that in experimental NTS nephritis, JQ1 decreased kidney *Grem-1* and *Ccl8* expression, and this was associated to milder macrophage cell infiltration and the amelioration of kidney damage, therefore supporting their potential use as biomarkers of disease progression.

The role of the NOTCH pathway in kidney inflammation and CKD progression has been extensively described [28,33,34]. NOTCH activation has been described in several human glomerulopathies, including cGN [23,24,25,26,27,28]. In rat anti-GBM rapidly progressive GN, *Notch3* and *Hey-1* mRNAs were upregulated [63]. In murine NTS-nephritis investigated here, *Hes-1* and *Hey-1* mRNAs were overexpressed at 10 days. Several data suggest that BET proteins can interact with the NOTCH pathway. In cancer cells, BRD4 can bind to Jagged-1 and NOTCH1 promoters, increasing the gene expression of both NOTCH components and thus leading to NOTCH pathway activation. By this mechanism, the proliferation and migration in tumors is promoted [64,65]. In addition, I-BET151 impeded BRD4 binding to the NOTCH1 promoter, diminishing the expression of *Hes1* and cancer-cell renewal activity [64,65]. Accordingly, we observed that JQ-1 downregulated *Hes-1* and *Hey-1* expression in NTS mice. In tumor cells, c-MYC directly activates RBPJκ and subsequent target genes in a NOTCH activation-independent manner, mediated by CDK9, a component of positive transcription elongation factor b (P-TEFb). Treatment with JQ-1 in these cells deregulated the gene expression of *Hes-1* and *Hey-1*, maybe via a CDK9–MYC–RBPJ pathway [66]. In addition, the interaction between the NOTCH and NF-κB pathways contributes to tissue damage progression [67,68]. Importantly, the activation of both signaling pathways can be targeted by iBETs. In experimental kidney disease, treatment with the NOTCH inhibitor DAPT blocked NF-κB-mediated inflammation [20]. Mice lacking *Notch3* expression exhibited lower NF-κB activation in glomeruli associated to milder proteinuria, uremia and inflammatory infiltration [63]. iBETs inhibit NF-κB activation by binding to the acetylated lysine-310 of RelA/p65 NF-κB in the nuclei, leading to the ubiquitination and degradation of the constitutively active nuclear form of RelA/NF-κB [10,69,70,71]. Since Gremlin-1 activates NF-κB and NOTCH pathways in the kidney [20], we postulate that the JQ1-mediated decrease in *Grem-1* transcription could also potentially inhibit the Gremlin-mediated activation of both NF-κB and NOTCH pathways, and therefore, their contribution to CKD progression. Although future studies evaluating the potential effects of iBETS on NOTCH signaling activation in kidney damage are needed, our findings support the notion that iBETs can modulate NOTCH pathway activation by different molecular mechanisms.

Podocyte loss is a feature of progressive GN [72], as we have observed in experimental NTS nephritis. Interestingly, JQ1 treatment restored podocyte numbers in injured kidneys. In response to damage, podocytes lose their properties, including phenotype changes, leading to increased production of cytokines, growth factors and extracellular matrix components and, finally, to podocyte cell death [72,73]. NOTCH activation in mature podocytes can induce apoptosis [74,75,76]. The conditional deletion of RBPJ specifically in podocytes in mice with diabetic nephropathy was associated with reduced podocyte apoptosis [74]. In cultured podocytes, the overexpression of active Notch3 led to cytoskeleton reorganization and changes to a proliferative/migratory and inflammatory phenotype [63]. In a model of conditional WT1 deletion in mature podocytes, the upregulation of several NOTCH pathway components, including Hes, was associated with the upregulation of genes implicated in phenotype changes [77]. These evidences suggest that inhibition of NOTCH/Hes by JQ1 could be involved in the observed beneficial effects on podocyte number preservation in experimental NTS nephritis.

Preclinical data suggest that iBETs can also diminish experimental fibrosis, as described in bleomycin- and radiation-induced lung fibrosis [78,79,80] and in diabetic cardiomyopathy [81]. Interestingly, integrated transcriptomics analyses across animal models of cardiac damage and human cells have found that iBETs preferentially inhibited genes of the innate inflammatory and profibrotic myocardial pathways, mainly of the NF-κB and TGF-β signaling networks [82]. Regarding experimental CKD, JQ1 decreased fibrosis in the UUO model [83,84]. Now, the data presented here show that JQ1 diminished *Grem-1* overexpression in obstructed kidneys of UUO mice. Gremlin-1 has been involved in several fibrotic disorders. In vitro, Gremlin-1 promotes fibroblasts proliferation and extracellular matrix production. Moreover, Gremlin-1 can induce the partial epithelial-to-mesenchymal transition of cultured tubular epithelial cells by activating Smad [85] and NOTCH signaling pathways [22], suggesting a potential role of Gremlin-1 in kidney fibrosis. In human ANCA-associated cGN, the overexpression of Gremlin-1 correlated with tubulointerstitial fibrosis [39], suggesting that this factor can also be involved in tubulointerstitial fibrosis in this pathology. Although a limitation of the present study is that the murine NTS model studied here does not present tubulointerstitial fibrosis at the time-point studied, our findings showing that JQ1 inhibited *Grem-1* expression in the UUO model support that the beneficial effects of iBETs can also be mediated by the inhibition of Gremlin-1-induced profibrotic events in the kidney.

Our studies using a small-molecule inhibitor targeting bromodomain proteins highlight the functional importance of bromodomain/acetyl-lysine binding as a key mechanism in orchestrating molecular interactions and regulation in chromatin biology and gene transcription. Clinical studies inhibiting BET proteins or the NOTCH system have so far focused on cancer or cardiovascular disease [86]. Actually, there are several clinical trials about the role of iBETs in tumoral diseases such as solid tumors (ODM-207: NCT03035591 and ABBV-075: [87]); Metastatic Castration-resistant Prostate Cancer (ZEN-3694; [88]); refractory non-Hodgkin’s lymphoma (CC-90010: NCT03220347); diffuse large B-cell lymphoma (RO6870810: NCT01987362); acute leukemia and multiple myeloma (OTX015: NCT01713582; RO6870810: NCT02308761 and CPI-0610:NCT02157636). In patients with cardiovascular diseases such as atherosclerosis (ASSURE Trial/RVX-208: NCT01067820) or acute coronary syndrome (BETonMACE trial/Apabetalone: NCT02586155), iBETs have shown beneficial effects. In kidney diseases there is a recent open phase II study in Fabry disease to evaluate the role of RVX000222 in cardiovascular damage markers, including alkaline phosphatase levels and calcification markers, such as RANKL (NCT03228940). Our findings showing that JQ1 inhibited *Grem-1* and the gene transcription of NOTCH-effectors (*Hes-1* and *Hey-1)* in experimental NTS nephritis, together with previous studies showing inhibition NF-κB/proinflammatory genes (as now also shown for *Ccl8*) [32], support further research on epigenetic drugs, such as iBETs, for rapidly progressive cGN.

## 4. Materials and Methods

### 4.1. Ethics Statement

All animal procedures were performed in 3-month-old C57BL/6 mice. Surgeries were performed under isoflurane-induced anesthesia, according to the guidelines of animal research in the European Community and with prior approval by the Animal Ethics Committee of the Health Research Institute IIS-Fundación Jiménez Díaz.

### 4.2. Experimental Models

The BET bromodomain inhibitor JQ1, a thieno-triazolo-1,4-diazepine, was synthesized and provided collaboratively by Dr. James Bradner (Dana-Farber Cancer Institute, Boston, MA, USA) [89]. For in vivo studies, JQ1 was dissolved in 10% hydroxypropyl β-cyclodextrin and used at a therapeutic dose (100 mg/kg body weight per day, i.p.), as previously described [32].

Anti-murine glomerular basement membrane (GBM) nephritis was induced in male C57BL/6 mice by administering rabbit nephrotoxic serum (NTS) as described [30]. Mice were injected with 50 μL of NTS diluted 1/10 in sterile saline on day 1. Then, 4 μL/g of body weight were injected on day 2 and day 3, and mice were studied 10 days later. Mice used were 7 NTS and 7 NTS treated with JQ1 and 5 controls (*n* = 5−7 mice per group). Some animals were also treated daily with JQ1 or vehicle starting one day before the first NTS administration.

For unilateral ureteral obstruction (UUO), the left ureter was ligated with silk (5/0) at two locations and cut between ligatures to prevent urinary tract infection (obstructed kidney) in male C57BL/6 mice, under isoflurane-induced anesthesia, as described [22]. Some animals were treated daily with JQ1 from 1 day before UUO and studied after 5 days (*n* = 6−7 mice per group). At the time of sacrifice, animals were anesthetized with 5 mg/kg xylazine (Rompun, Bayer AG, Leverkusen, Germany) and 35 mg/kg ketamine (Ketolar, Pfizer, Brooklyn, NY, USA), and the kidneys were perfused in situ with cold saline before removal. Kidneys were further processed for immunohistochemistry (fixed and paraffin-embedded) and ChIP assays (fixed in 1% formaldehyde followed quenching with 0.125 M glycine) or snap-frozen in liquid nitrogen for RNA and protein studies.

### 4.3. Histology and Immunohistochemistry

Paraffin-embedded kidney sections were stained using standard histology procedures as described elsewhere [32]. Kidney injury was evaluated by Masson staining. 

Immunostaining was carried out in 3 μm thick tissue sections. Antigen retrieval was performed using the PTlink system (Dako, Glostrup, Denmark) with sodium citrate buffer (10 mM) adjusted to pH 6–9, depending on the immunohistochemical marker. Endogenous peroxidase was blocked. Tissue sections were incubated for 1 h at room temperature with 4% BSA and 10% of a specific serum (depending on the secondary antibody used) in PBS to eliminate non-specific protein binding sites. Primary antibodies were incubated overnight at 4 °C. Specific biotinylated secondary antibodies (Amersham Biosciences, Amersham, UK) were used, followed by streptavidin–horseradish peroxidase conjugate and 3,3-diaminobenzidine as a chromogen. The primary antibodies used were: F4/80 [1:50 MCA497, Bio-Rad, Hercules, CA, USA] and WT-1 [1:100 M3561, Dako]. Specificity was checked by omission of primary antibodies. Quantification was made by determining in 5 to 10 randomly chosen fields (200× magnification) the total number of positive cells using Image-Pro Plus software (data expressed as the positive-stained area relative to the total area) or quantifying manually the number of positive nuclei.

### 4.4. Gene Expression Studies

RNA from cells or renal tissue was isolated with TRItidy G^TM^ (PanReac, Barcelona, Spain). cDNA was synthesized by a High Capacity cDNA Archive kit (Applied Biosystems, Waltham, MA, USA) using 2 μg total RNA primed with random hexamer primers. Next, quantitative gene expression analysis was performed by real-time PCR on an AB7500 fast real-time PCR system (Applied Biosystems) using fluorogenic TaqMan MGB probes and primers designed by Assay-on-DemandTM gene expression products. Mouse assays IDs were: *Arg2*: Mm00477592_m1, *Cd163*: Mm00474091_m1, *Ccl8*: Mm01297183_m1, *Cxcl2*: Mm00436450_m1, *Dlk1*: Mm00494477_m1, *Gremlin-1:* Mm00483888_s1, *Hes-1*: Mm01342805_m1, *Hey-1*: Mm00468865_m1, *Il10*: Mm00439616_m1, *Jagged-1:* Mm00496902, *Notch1*: Mm00435249_m1, *Notch2*: Mm00803077_m1, *Notch3*: Mm01345646_m1, *Notch4*: Mm00440525_m1, *Pai-1*: Mm00435858_m1 and *Tgf-β*: Mm01178820_m1. Data were normalized to *Gapdh*: Mm99999915_g1 (Vic). The mRNA copy numbers were calculated for each sample by the instrument software using Ct value (“arithmetic fit point analysis for the lightcycler”). Results were expressed in copy numbers, calculated relative to unstimulated cells.

### 4.5. Protein Studies

Total protein samples for frozen renal tissue were isolated in lysis buffer (50 mmol/L Tris-HCl, 150 mol/L NaCl, 2 mmol/L EDTA, 2 mmol/L EGTA, 0.2% Triton X-100, 0.3% IGEPAL, 10 μL/ml proteinase inhibitor cocktail (CIP), 0.2 mmol/L PMSF, and 0.2 mmol/L orthovanadate) as described [32]. Proteins (20-100 μg per lane, quantified using a BCA protein assay kit) were separated on 8–12% polyacrylamide-SDS gels under reducing conditions [32]. For Western blotting, cell (50 μg/lane) protein extracts were separated on 8%–12% polyacrylamide-SDS gels under reducing conditions. Samples were then transferred onto polyvinylidene difluoride membranes (Thermo Scientific, Waltham, MA, USA), blocked with TBS/5% non-fat milk/0.05% Tween-20 and incubated overnight at 4°C with the following antibody (dilution): Gremlin-1 (1:1000; Sc-515877; Santa Cruz). Membranes were subsequently incubated with peroxidase-conjugated IgG secondary antibody and developed using an ECL chemiluminescence kit (Amersham, Amersham, UK). Loading controls were performed using an anti-GAPDH antibody (1:5000; CB1001, Millipore). Results were analyzed by LAS 4000 and Amersham Imager 600 (GEHealthcare, Chicago, IL, USA) and densitometered by Quantity One software (Biorad, Hercules, CA, USA). The evaluation of CCL8 in kidney tissue was performed by ELISA (R&D system, Cat. No. DY790) following the instructions provided by the manufacturer.

### 4.6. Chromatin Immunoprecipitation

Kidneys were fixed in 1% formaldehyde (Sigma-Aldrich, St. Louis, MO, USA) followed by quenching with 0.125M glycine. DNA fragments 500–1000 bp long were generated on a BioRuptor (Diagenode), and ChIP assays were performed using the High Cell ChIP kit (Diagenode, Denville, NJ, USA) following the manufacturer’s instructions. The antibodies used were BRD4 (Bethyl Laboratories, Burlington, MA, USA) and normal IgG as negative control (Millipore). Immunoprecipitated DNA was analyzed by quantitative RT-PCR using the following primers of *Grem-1* promoter:

- Forward 5′-GACCAATGGAGAGACGCAGT-3′
- Reverse 5′-GTTCTTCGCTGTGGACGAGT-3′

Chromatin obtained before immunoprecipitation was used as the input control. Relative enrichment was calculated as the percentage of input DNA for each sample using the formula % input = 2 exp [(Ct unbound) − log_2_ (unbound dilution factor) – Ct bound)] × 100 and normalized to normal rabbit IgG antibody (considered as 1).

### 4.7. Statistical Analysis

Results are expressed as mean ± SEM of the *n*-fold increase with respect to the control (represented as 1). In the NTS nephritis model, data were obtained normalizing NTS and NTS +JQ1 kidneys versus control average. In the UUO model, data were obtained comparing obstructed kidneys versus untreated contralateral kidneys (represented as 1). The Shapiro–Wilk test was used to evaluate sample normality distribution. If the samples followed the Gaussian distribution, a one-way ANOVA followed by the corresponding post-hoc analyses of Fisher’s LSD test were used. To compare non-parametric samples, a Kruskal–Wallis and a subsequent post-hoc analysis of Uncorrected Dunn’s test was performed. Statistical analysis was conducted using GraphPad Prism 8.0 (GrahPad Software, San Diego California United States). Values of *p* < 0.05 were considered statistically significant.

## 5. Conclusions

Nowadays, the current clinical option for ANCA^+^ GN is immunosuppressive treatment, but this therapy only slows the progression of kidney damage, and most ANCA patients progress to ESRD, being a serious threat to survival. Hence, the search for novel new therapeutic strategies aims to slow its progression or even revert this lesion is an unmet and an urgent necessity. Clinical trials using iBETs have shown some beneficial effects in different diseases including kidney diseases. Our preclinical data showing that Gremlin-1, a proposed biomarker of disease activity in ANCA patients, is as a specific target of BET inhibition further support the investigation of iBETs as a therapeutic option for human cGN.

## Data Availability

The data are contained within the article.
